# Extreme Elevations in Blood Pressure and All-Cause Mortality in a Referred CKD Population: Results from the CRISIS Study

**DOI:** 10.1155/2013/597906

**Published:** 2013-04-22

**Authors:** James Ritchie, Francesco Rainone, Darren Green, Helen Alderson, Diana Chiu, Rachel Middleton, Donal O'Donoghue, Philip A. Kalra

**Affiliations:** Vascular Research Group, Department of Renal Medicine, Salford Royal Hospital, Salford M6 8HD, UK

## Abstract

Hypertension frequently complicates chronic kidney disease (CKD), with studies showing clinical benefit from blood pressure lowering. Subgroups of patients with severe hypertension exist. We aimed to identify patients with the greatest mortality risk from uncontrolled hypertension to define the prevalence and phenotype of patients who might benefit from adjunctive therapies. 1691 all-cause CKD patients from the CRISIS study were grouped by baseline blood pressure—target (<140/80 mmHg); elevated (140–190/80–100 mmHg); extreme (>190 and/or 100 mmHg). Groups were well matched for age, eGFR, and comorbidities. 77 patients had extreme hypertension at recruitment but no increased mortality risk (HR 0.9, *P* = 0.9) over a median follow-up period of 4.5 years. The 1.2% of patients with extreme hypertension at recruitment and at 12-months had a significantly increased mortality risk (HR 4.3, *P* = 0.01). This association was not seen in patients with baseline extreme hypertension and improved 12-month blood pressures (HR 0.86, *P* = 0.5). Most CKD patients with extreme hypertension respond to pharmacological blood pressure control, reducing their risk for death. Patients with extreme hypertension in whom blood pressure control cannot be achieved have an approximate prevalence of 1%. These patients have an increased mortality risk and may be an appropriate group to consider for further therapies, including renal nerve ablation.

## 1. Introduction

The global epidemic of chronic kidney disease (CKD) represents a significant challenge for healthcare providers [1]. Despite ever-increasing numbers of patients identified with CKD, there is a paucity of evidence to accurately describe outcomes and optimal management strategies for this high-risk population. Consequently, many therapeutic decisions are extrapolated from studies performed in the general population. This may be inappropriate as many characteristics well recognized as risk factors for mortality in the general population exhibit reverse epidemiology in the CKD or dialysis population [2, 3]. Hypertension is one such example where patients with CKD can differ from the general population in terms of morbidity/mortality risk and benefit from treatment [4, 5].

Hypertension and CKD are inextricably linked with both cause and effect relationships. Uncontrolled blood pressure is associated with a more rapid loss of estimated glomerular filtration rate (eGFR) [6]. As such, aggressive treatment of hypertension has been a key component of CKD management for many years [7]. Despite this clinical focus, the evidence of benefit from such stringent blood pressure control is perhaps less concrete than it is perceived to be. Although strict blood pressure control has been shown to reduce the rate of CKD progression, the evidence for this is much stronger in the pediatric than adult CKD population. Evidence of reduced mortality/cardiovascular events with “optimal” blood pressure management is even more limited [8, 9]. This can be partly rationalized given that marked baseline hypertension appears to have only a modest effect on risk for death in predialysis CKD [10] and is often found to be a less important adverse prognostic marker than hypotension [11]. It may be that the important pathophysiological changes to the vasculature (and subsequent risk) associated with CKD relate more to vascular calcification than a blood pressure mediated process [12].

Despite these gaps in our knowledge, hypertension in CKD continues to be a focus for investigation. Much interest currently surrounds renal sympathetic nerve ablation—a technique shown to have significant effects on systolic and diastolic blood pressures in both the general and CKD populations with resistant hypertension [13–15]. However, despite the very positive findings in terms of blood pressure control, no outcome data has been published to show an effect upon hard clinical end-points such as mortality. To design a meaningful interventional study it is vital to accurately identify the CKD patients with the highest risk for death in relation to elevated blood pressure. In this study we attempt to define the phenotype and prevalence of patients who might benefit from newer adjunctive therapies that lower blood pressure. 

## 2. Materials and Methods

The study population was drawn from patients recruited to the Chronic Renal Insufficiency Standards Implementation Study (CRISIS) prior to 31 January 2010. Approval for this study was granted by the regional ethics committee and all patients provided full written informed consent. Details of CRISIS have been published previously [16]; in brief this is a prospective observational study of outcomes (death and renal replacement therapy) in an all-cause CKD population. All patients aged 18 years and over referred to our tertiary nephrology center (catchment population 1.55 million) with an eGFR <60 mL/min/1.73 m^2^ and not requiring immediate referral for dialysis are approached for consent. Baseline demographic data (age, gender, ethnicity, smoking history, cause of CKD, and comorbid conditions) are recorded, as are annual measurements of blood pressure, prescribed medications, and laboratory data (eGFR, proteinuria, and hemoglobin). Mortality data are obtained from the Office of National Statistics. All blood pressure measurements are made by trained staff in accordance with trust protocol. An automated sphygmomanometer with an appropriately sized cuff is used, with all measurements made after at least 5 minutes of seated rest. Patients are requested not to consume caffeine alcohol or undertake vigorous exercise prior to clinic visits. A minimum of two readings are obtained, with an average of these results recorded. For this analysis, patients were grouped into categories of blood pressure:

1. target: systolic blood pressure <140 mmHg and diastolic blood pressure <80 mmHg. This group was used as the referent category;
2. elevated: systolic blood pressure 140–190 mmHg and/or diastolic 80–100 mmHg;
3. extreme hypertension: systolic blood pressure >190 mmHg or diastolic >100 mmHg. Although these values differ from classical definitions of, for example, stage III hypertension, they were selected as they identified the 5% of the study population with the highest baseline blood pressures and in part reflect the more rapid increases in vascular stiffness observed in CKD [17].

Normally distributed data are presented as mean ± standard deviation, with non-parametric data presented as median (interquartile range). Survival analysis was performed using multivariate Cox proportional hazards regression with a forward stepwise methodology (*P* for inclusion and retention <0.3). Censoring occurred at death, last clinic visit, or 1 July 2012. Unless specified in the text, all presented hazard ratios are from multivariate analysis, with results presented as hazard ratio (95% confidence interval). Event rates were compared using negative binomial regression and correlations between variables assessed with Pearson's correlation coefficient. Unless otherwise stated, statistical significance was defined as *P* < 0.05. All analyses were performed in SAS version 9.2 (SAS Institute, Cary, NC, USA) under license to the University of Manchester (UK). 

## 3. Results

### 3.1. Patient Characteristics

1750 patients were recruited to CRISIS prior to 31 January 2010, with complete baseline data available for 1691 patients, who formed the study population. At recruitment, median patient age was 67 years (IQR 55–75), mean eGFR 32 ± 15 mL/min/173 m^2^, and mean blood pressure 137/75 mmHg. Median followup was 4.5 years (IQR 2.9–6.9 years). When grouped according to baseline blood pressure, 722 (42%) had target blood pressure, 892 (53%) had elevated blood pressure, and 77 (5%) had extreme hypertension. In the extreme hypertension group, 45% of patients met the systolic blood pressure definition, 71% of patients met the diastolic blood pressure definition, and 16% of patients met both definitions. Primary cause of CKD was well matched between groups. In comparison between all groups, baseline characteristics were well matched between all three blood pressure groups, with significant differences only observed in urinary protein to creatinine ratio, which increased with blood pressure group (target, elevated, extreme; 84 ± 146, 117 ± 213, 104 ± 148 mg/mmol, *P* = 0.005); hemoglobin, which also increased (122 ± 16, 125 ± 17, 131 ± 19 g/L, *P* = 0.003); and history of myocardial infarction, which was inversely associated with increasing blood pressure (20%, 17%, 10%, *P* = 0.045). Complete baseline data are presented in Table 1. 

### 3.2. Associations between Baseline Blood Pressure and Mortality

When considered as a continuous variable, neither baseline systolic, or diastolic blood pressure was associated with a significant change in hazard ratio (HR) for death within 12 months of recruitment. Both, however, had minor associations with risk for mortality over the complete follow-up period (HR for death per mmHg increase: SBP 1.003 [1.0–1.007]; DBP 0.98 [0.97–0.99], *P* for both <0.01). 

When patients were considered by group of baseline blood pressure, there was a nonsignificant trend towards increased risk for death within 12 months for patients in the extreme hypertension group (HR 2.4 [0.9–6.7], *P* = 0.09). However, there was no association between baseline group of blood pressure and risk for mortality over the entire follow-up period, with neither baseline elevated blood pressure nor extreme hypertension reaching sufficient statistical significance to enter the multivariate model (unadjusted HR 0.86 [0.7–1.0] and 0.94 [0.7–1.3], resp., *P* > 0.05). Complete data are presented in Table 2. 

### 3.3. Associations between Extreme Hypertension Persisting at 1 Year and Mortality

Of the 77 patients in the extreme hypertension group at baseline, 5 (6.5%) died within the first 12 month followup. Of the 72 survivors at 1 year, only 9 (12.5%) still met the criteria to be classified as having extreme hypertension despite being prescribed more antihypertensive medications than the 55 patients with a documented blood pressure who had transferred into a lower category of blood pressure (mean number of antihypertensive medications at 1 year 3.4 versus 2.6, *P* = 0.3). Patients who continued to be classified as having extreme hypertension had significantly higher blood pressures than those no longer classified as such (175/109 mmHg versus 141/78 mmHg, *P* < 0.001). 

In the 9 patients where extreme blood pressure elevations persisted at 1 year, a significant increase in risk for death was observed (overall mortality 55%, median time to death 3.5 years (IQR 1.8–5.9)). This increase was relative both to the patients who initially, but no longer, had extreme hypertension (overall mortality 40%, median time to death 4.8 years IQR 2.3–6.3), with an HR for death 3.47 [1.1–11.0], *P* = 0.03, and also to the population who was not classified as having extreme hypertension at baseline (overall mortality 39%, median time to death 4.7 years IQR 2.9–6.8), HR for death 4.3 [1.5–12.7], *P* = 0.001. Notably, the patients who were no longer classified as having extreme hypertension at 1 year did not have an increase in risk for death compared to the remainder of the study population (unadjusted HR 0.86 [0.6–1.3], *P* = 0.5). These data are presented in Table 3. 

No significant differences in the distribution of baseline antihypertensive medications were observed between patients classified as having extreme hypertension and the remainder of the study population. The same overall finding was replicated when medication use at 12 months was considered, although here a trend towards higher rates of diuretic use in patients with persistent extreme hypertension was observed (55% versus 24%,  *P* = 0.1). Complete data on antihypertensive medication use are presented in Table 4. 

### 3.4. Associations between Development of Extreme Hypertension at 1 Year and Overall Mortality

A separate analysis was performed for patients with target or increased baseline blood pressure but who then went on to develop extreme hypertension during followup. Of the 1614 patients without baseline extreme hypertension, 1 year follow-up data were available for 1332 (82.5%)—91 dead and 191 discharged or lost to followup. Of these 1332 patients, 602 were in the target blood pressure group at baseline (60% remained in the target group; 38% moved to the elevated blood pressure group; 2% moved to the extreme hypertension group) and 730 patients were in the elevated blood pressure group at baseline (57% of which remained in this group, 36% moved down to the target blood pressure group, and 6% moved to the extreme group). Overall 58 (4.4%) patients had sufficient increase in their blood pressure to move into the extreme hypertension category during followup. 

Movement into the extreme blood pressure group at 1 year was not associated with an increased risk for death either throughout the complete followup (unadjusted HR 1.03 [0.8–1.5], *P* = 0.9) or in the subsequent 12 months (unadjusted HR 1.31 [0.8–1.7], *P* = 0.2). Of the 58 patients who moved into the extreme hypertension group at 12 months, 24-month follow-up data were available for 45, with 6 of these patients continuing to be classified as having extreme hypertension and 39 having moved down to a lower blood pressure group. No significant increased risk for death was observed in the 6 patients who remained in the extreme hypertension group either when compared to the 39 patients who had moved to a lower category of blood pressure (unadjusted HR 1.1 [0.2–4.3], *P* = 0.8) or the remainder of the population (unadjusted HR 0.96 [0.3–3.1],  *P* = 0.9). Overall blood pressure trends are described in Table 5. 

## 4. Discussion

Although limited by a lack of data regarding medication doses and patient compliance, and acknowledging that blood pressure data was obtained from a limited number of time points, these analyses have identified several findings relevant to future study design. Firstly, we have demonstrated that only a small proportion of patients in a referred secondary care nephrology population, 20 (1.2%) in this analysis, has extreme elevations in blood pressure that persist despite specialist intervention. This may have implications when designing the studies needed to compare hard clinical end-points between novel interventional and standard pharmacological therapies. Furthermore, this study raises questions about the value of considering hypertension in patients with moderate to advanced chronic kidney disease in a categorical manner. We have demonstrated an association between baseline blood pressure and mortality in an all-cause CKD population. This is consistent with previous studies that have shown that coexistent renal impairment is an independent risk factor for mortality for hypertensive patients [18] and that lowering blood pressure in CKD can reduce risk for cardiovascular mortality [19]. However, an important negative finding is the lack of a significant association between baseline blood pressure group and risk for death. Whilst we accept that our defined blood pressure groups are not directly comparable to the Symplicity studies [13], our results suggest that without further work to delineate a high-risk patient group (within the already high-risk CKD population) any future study may fail to identify the mortality benefit that would be anticipated given the blood pressure reduction observed following renal sympathetic nerve ablation, that is, risk of a type-2 error. 

Secondly, we have demonstrated that extremes of blood pressure are not restricted to patients with the most advanced CKD. Whilst an inverse relationship between eGFR and systolic blood pressure existed (Pearson's correlation coefficient  −0.06, *P* = 0.01), consistent with other studies in CKD populations [20], baseline eGFR did not differ significantly between blood pressure groups. Other patient characteristics, however, did differ, with hemoglobin levels significantly higher in the extreme hypertension group. Whilst many CKD patients are treated with erythropoiesis stimulating agents (ESA), a recognized side effect of which can be elevated blood pressure [21], equal proportions of patients in each group had a baseline history of ESA use (13%, 14%, 14%, resp., *P* = 0.6). This equal distribution has further relevance, given the potential links between these agents and an increased risk for death [22]. More important perhaps was the difference in history of myocardial infarction between groups. Patients in the lowest blood pressure group had a significantly greater history of documented myocardial infarction (20% versus 10% in the EH group). Without supporting echocardiographic data, we cannot be certain that outcomes for the lowest or target group of blood pressure were not confounded by higher rates of systolic dysfunction—a strong predictor of outcome in the CKD population [23]. 

Our most novel findings relate to patients with “extreme hypertension” at baseline. Although this subgroup was limited in patient numbers, there was a clear signal towards increased short-term but not long-term mortality in this cohort. We suggest that this related to significant improvements in blood pressure control in the majority of these patients in the 12 months following recruitment. By one year, 87% of surviving patients classified as having extreme hypertension at baseline no longer fitted in this category. These patients saw a mean blood pressure reduction of 36/23 mmHg in comparison to the 9 patients with persistent extreme blood pressure, who saw a mean change of 0/3 mmHg. These vast differences in blood pressure control related only to a modest increase in number of antihypertensive agents and small increases in the number of patients prescribed either angiotensin blockade or calcium channel blockers. As such, the most logical conclusions are that either dosing alterations or improved compliance facilitated the improved control. This highlights the benefits of specialist care given that patients with CKD are often underdosed and undertreated for fear of worsening renal function [24, 25]. Given the restrictions of the data available we are unable to comment upon other possible mechanisms of blood pressure reduction including dietary salt restriction, weight loss, and reduced alcohol consumption; as such the possible confounding effects of these interventions should not be discounted. The prognostic importance of achieving such improvements in blood pressure is thrown into stark relief when the large increases in risk for death for patients who had persistent extreme hypertension at 1 year are considered. All patients at our center are treated in line with national guidance on blood pressure targets [7] and returned for at least one follow-up visit. Hence it is highly unlikely that the lack of improvement in blood pressure for these 9 patients represents either undertreatment or complete patient disengagement with healthcare. Equally, it is unlikely that failure to manage blood pressure represented an undiagnosed cause of secondary hypertension. Of the 9 patients, the majority had documented evidence of investigation to exclude renal artery stenosis (either bilaterally normally sized kidneys on ultrasound or indirect angiography) and investigation to exclude Conn's syndrome. Also, the differences in prescribed medications between recruitment and 12 months are consistent with active management. Despite this, a 4-fold increased risk for death existed, suggesting that this patient group is potentially an important one to consider in future interventional studies of blood pressure management with a mortality end-point. Whilst the observational nature of these data preclude attribution of causality, the higher proportion of patients with persistent extreme hypertension prescribed diuretics is interesting. Although no distinction is drawn between types of prescribed diuretics (with thiazides potentially less effective antihypertensive agents where eGFR is <30 mL/min/1.73 m^2^), the secondary care setting of this study makes ineffective prescribing unlikely. Potentially, the increased use of these agents is representative of a higher proportion of patients with CKD-related salt/water retention. As such the elevations in blood pressure may represent a volume-mediated process compounding increased systemic vascular resistance. As fluid overload in CKD has been associated with increased risk for death [26, 27], this may be a relevant factor for future study. 

Finally, we have demonstrated that the risks of extreme elevations in blood pressure appear to vary over time. Although it initially seems incongruous that patients with normal or moderately raised blood pressure at baseline who transit into the category of extreme hypertension do not have the same increased risks for death as patients with extreme hypertension at baseline, this may relate to several factors in addition to the small sample size in these categories. Firstly, it is highly probable that patients with extreme blood pressure at time of referral have been exposed to this vascular risk for a substantial period of time, whilst those who develop extreme hypertension during followup can be more readily identified and quickly treated. Secondly, increases in blood pressure during followup were strongly related to reductions in eGFR (Pearson's correlation coefficient 0.15, *P* < 0.0001), with a signal towards greater percentage of 2-year reductions in eGFR observed in patients moving into the extreme hypertension group versus those who remained in the target or elevated groups (12% versus 8% reduction, *P* = 0.6). Hence patients developing extreme hypertension during followup may have suffered the greatest decline in renal function. Given that renal function is one of the strongest predictors of mortality in CKD [28], it is possible that the risks associated with increases in blood pressure over 1 year are outweighed by the risks associated with risk of a reduced eGFR. 

This analysis has several limitations. Firstly, the small patient numbers in some groups may have implications for model stability; as such the reproducibility of these findings needs to be assessed in another cohort. Secondly, due to the observational nature of this study, no data was available regarding compliance with medications, medication dosing, or dietary sodium restriction. Finally, despite the increased 12 month mortality risk shown in the “extreme hypertension” group, this study cannot directly answer the question of which subgroup of hypertensive CKD patients would be of greatest interest in a future study of interventional blood pressure therapy. 

In conclusion, this study suggests that failure to manage extreme elevations in blood pressure is more important than absolute baseline blood pressure as a predictor of mortality in CKD. Of the 77 patients with extreme hypertension at baseline, 14 either died within the first 12 months or had persisting extreme hypertension at this time. Another 6 patients progressed to develop persistent extreme hypertension from a less severe baseline category. Hence 1.2% patients in this secondary care CKD population in whom extreme elevations in blood pressure persisted despite specialist care would warrant further study both to understand why blood pressure cannot be controlled and also to identify their suitability for interventions that may mitigate their high adverse risk. This provides an estimate of the proportion of a CKD population that might be suitable for interventional techniques to lower blood pressure, such as renal nerve ablation therapy.

## Figures and Tables

**Table 1:** Baseline patient characteristics divided by blood pressure group.

	Group 1—target blood pressure *n* = 722	Group 2—elevated blood pressure *n* = 892	Group 3—extreme hypertension *n* = 77	*P*
Age	64.9 (14.2)	63.1 (36.6)	63.4 (14.2)	0.43
Weight	80.5 (18.3)	81.6 (17.9)	80.4 (19.6)	0.4
Systolic blood pressure (mmHg)	120.2 (12)	146.7 (16)	178.5 (23.3)	<0.0001
Diastolic blood pressure (mmHg)	66.7 (7.6)	79.3 (9.9)	101 (14.4)	<0.0001

	Laboratory values			

eGFR (mL/min/1.73 m^2^)	33.6 (15.4)	32.1 (16.1)	33.9 (18.7)	0.25
Urine protein creatinine ratio (mg/mmol)	83.6 (146.4)	116.7 (213.3)	104.1 (147.7)	0.005
Haemoglobin (g/dL)	122.6 (16.7)	125.4 (17.1)	130.7 (19.2)	0.003

	Medications			

Number of antihypertensive agents	2.4 (1.4)	2.4 (1.4)	2.5 (1.5)	0.81
Angiotensin blockade	63.2%	59.4%	55.8%	0.24
Statin	60.4%	57.3%	49.4%	0.10
Aspirin	42.7%	39.5%	36.4%	0.25
Erythropoietin stimulating agent	12.8%	14.5%	14.3%	0.62

	Co-morbidities			

Myocardial infarction	20.4%	16.8%	10.4%	0.045
Transient ischemic attack or stoke	15%	16.1%	19.5%	0.56
Diabetes mellitus	29.8%	25%	26%	0.097
Smoking history	69.5%	65.9%	72.7%	0.15

	Primary cause of CKD			

Diabetic nephropathy	88 (12.2%)	116 (13%)	8 (10.4%)	0.88
Adult polycystic kidney disease	30 (4.2%)	50 (5.6%)	4 (5.2%)	0.43
Vascular/hypertensive	104 (16.4%)	137 (19.0%)	1 (17.5%)	0.44
Glomerulonephritis/vasculitis	89 (14.0%)	114 (15.7%)	14 (22.2%)	0.19
Pyelonephritis	37 (5.8%)	47 (6.5%)	3 (4.8%)	0.78
Other/unknown	298 (46.9%)	286 (39.7%)	23 (36.5%)	0.02

Continuous variables are presented as mean (standard deviation). Categorical variables are presented as number (percentage).

eGFR: estimated glomerular filtration rate (MDRD 4-variable formula). CKD: chronic kidney disease.

Target blood pressure defined as systolic blood pressure <140 mmHg and diastolic blood pressure <80 mmHg.

Elevated blood pressure defined as systolic 140–190 mmHg or diastolic 80–100 mmHg.

Extreme blood pressure defined as systolic >190 mmHg or diastolic >100 mmHg.

**Table 2:** Hazard ratio for death for baseline blood pressure variables.

	12-month mortality		Overall mortality	
	Hazard ratio (95% confidence interval)	*P*	Hazard ratio (95% confidence interval)	*P*
Systolic blood pressure	Does not reach sufficient statistical significance for inclusion in model		1.003 (1.0–1.007)	0.007
Diastolic blood pressure	1.02 (0.99–1.04)	0.09	0.98 (0.97–0.99)	<0.001
Target blood pressure (*n* = 722)	Referent		Referent	
Elevated blood pressure (*n* = 892)	1.26 (0.7–2.2)	0.40	0.86 (0.7–1.0)*	0.06
Extreme hypertension (*n* = 77)	2.40 (0.9–6.7)	0.09	0.94 (0.7–1.3)*	0.9
Estimated glomerular filtration rate (mL/min/1.73 m^2^)	0.98 (0.97–1.0)	0.06
Hemoglobin (g/dL)	0.96 (0.95–0.98)	<0.0001
Angiotensin blockade	0.58 (0.3–0.9)	0.05
Statin	0.55 (0.31–0.97)	0.04
Myocardial infarction	3.70 (2.2–6.3)	<0.0001
Stroke or transient ischemic attack	2.15 (1.2–3.8)	0.009
Diabetes mellitus	1.51 (0.9–2.6)	0.1
Smoking history	1.82 (1.0–3.5)	0.06

Data are presented as hazard ratio (95% confidence interval).

Results for continuous variables are presented per 1 unit increment.

Angiotensin blockade defined as prescription of angiotensin converting enzyme inhibitor or angiotensin II receptor blocker.

Smoking history defined as current or previous smoking.

*does not reach statistical significance in multivariate analysis—value presented is unadjusted hazard ratio.

**Table 3:** Hazard ratio for death for patients with extreme hypertension at baseline.

	12-month mortality		Overall mortality	
	Hazard ratio (95% confidence interval)	*P*	Hazard ratio (95% confidence interval)	*P*
Resolved by one year (*n* = 55)	Referent		Referent	
Extreme hypertension persisting at one year (*n* = 9)	2.7 (0.5–13.7)	0.2	3.47 (1.1–11.1)	0.03
Age	1.06 (1.02–1.09)	0.002	1.05 (1.02–1.1)	0.05
Estimated glomerular filtration rate (mL/min/1.73 m^2^)	0.98 (0.96–1.01)	0.2	Not retained	
Hemoglobin (g/dL)	Not retained		0.96 (0.93–0.99)	0.05
Smoking history	Not retained		2.4 (0.8–7.4)	0.1
Diabetes mellitus	1.67 (0.7–3.9)	0.1	Not retained	

Data are presented as hazard ratio (95% confidence interval). Results for continuous variables are presented per 1 unit increment. Smoking history defined as current or previous smoking.

**Table 4:** Antihypertensive medications at baseline and one year.

	Baseline				1 year			
	Persistent extreme hypertension (*n* = 9)	Extreme hypertension at baseline, recovered by 1-year (*n* = 55)	Remainder of population (*n* = 1332)	*P*	Persistent extreme hypertension (*n* = 9)	Extreme hypertension at baseline, recovered by 1 year (*n* = 55)	Remainder of population (*n* = 1332)	*P*
Number of different antihypertensive medications	3.2 ± 1.5	2.4 ± 1.4	2.6 ± 1.3	0.19	3.4 ± 1.6	2.6 ± 1.3	2.7 ± 1.3	0.14
Angiotensin blockade	66%	58%	63%	0.73	78%	66%	65%	0.74
Diuretic	44%	26%	26%	0.45	55%	24%	24%	0.10
Calcium channel blocker	11%	14%	18%	0.68	11%	26%	18%	0.58
Beta blocker	22%	10%	10%	0.51	11%	12%	10%	0.94
Alpha blocker	0%	0%	6%	0.64	0%	0%	6%	0.64
Vasodilator	0%	0%	0.23%	0.93	0%	0%	0.23%	0.93
Centrally acting agent	10%	0%	0.4%	0.001	10%	0%	0.7%	0.001

Data are for patients with complete baseline and 1-year medication records.

**Table 5:** Long-term within-group changes in blood pressure.

		Year 0	Year 1	Year 2	Year 3	Year 4	Year 5
Target	Systolic blood pressure	120 (12)	131 (19)	133 (20)	132 (19)	135 (21)	136 (22)
	Diastolic blood pressure	67 (8)	72 (11)	72 (11)	70 (11)	73 (12)	72 (12)
	Total patient numbers	722	602	475	327	209	115
	Division by group (target/elevated/extreme)	722/0/0	359/231/12	272/191/12	196/127/4	101/103/5	57/55/3

Elevated	Systolic blood pressure	147 (16)	142 (22)	140 (22)	135 (20)	136 (21)	136 (22)
	Diastolic blood pressure	79 (10)	76 (12)	74 (12)	72 (11)	72 (12)	71 (12)
	Total patient numbers	892	730	595	452	309	222
	Division by group (target/elevated/extreme)	0/892/0	267/417/46	241/331/23	233/203/16	148/154/7	113/102/7

Extreme	Systolic blood pressure	179 (23)	146 (22)	144 (23)	138 (18)	144 (23)	138 (25)
	Diastolic blood pressure	101 (14)	83 (16)	80 (15)	78 (12)	75 (15)	73 (12)
	Total patient numbers	77	64	46	34	23	19
	Division by group (target/elevated/extreme)	0/0/77	16/39/9	15/24/7	14/19/1	9/12/2	12/7/0

Results are presented as mean (standard deviation).

Results show change within baseline blood pressure grouping over time.

Annual blood pressure values presented are those of surviving patients with follow-up data recorded at each individual time point.

Division by group describes the distribution of surviving patients with a documented blood pressure measurement between groups of blood pressure.
